# Development, validation and clinical impact of a prediction model for 6-month mortality in older cancer patients: the GRADE

**DOI:** 10.18632/aging.102876

**Published:** 2020-03-10

**Authors:** Eurydice Angeli, Kader Chouahnia, Florence Canoui-Poitrine, Boris Duchemann, Thomas Aparicio, Elena Paillaud, Laurent Zelek, Guilhem Bousquet, Frédéric Pamoukdjian

**Affiliations:** 1 APHP, Avicenne Hospital, Department of Medical Oncology, Bobigny F-93000, France; 2 INSERM, U942, Paris F-75010, France; 3 APHP, Henri-Mondor Hospital, Public Health Department, Créteil F-94000, France; 4 Université Paris-Est, UPEC, DHU A-TVB, IMRB- EA 7376 CEpiA (Clinical Epidemiology And Ageing Unit), Créteil F-94000, France; 5 Université Paris 13, Sorbonne Paris Cite, Villetaneuse F-93000, France; 6 APHP, Avicenne Hospital, Department of Gastroenterology, Bobigny F-93000, France; 7 APHP, Georges Pompidou European Hospital, Geriatric Department, Paris F-75015, France; 8 APHP, Avicenne Hospital, Geriatric department, Coordination Unit in Geriatric Oncology, Bobigny F-93000, France

**Keywords:** prognosis, decision support techniques, older adults, cancer, gait speed

## Abstract

Background: To develop, validate, and assess the clinical impact of a clinical score to predict a 6-month mortality risk among older cancer patients.

Results: The mean age was 81.2 ± 6.1 years (women: 54%, various cancers, metastatic cancer: 45%). The score, namely the GRADE, included two geriatric variables (unintentional weight loss, impaired mobility), two oncological variables (cancer site, cancer extension), and exclusively supportive care. Up to a 14% risk of early death, the decision curves suggest that cancer treatment should be instated.

Conclusion: We have developed and validated a simple score, easy to implement in daily oncological practice, to predict early death among older cancer patients which could guide oncologists in their treatment decisions.

Methods: 603 outpatients prospectively included in the Physical Frailty in Elderly Cancer patients cohort study. We created a multivariate prediction model by evaluating the strength of the individual components of the Geriatric Assessment regarding risk of death at 6 months. Each component was evaluated by univariate analysis and the significant variables (*P* ≤ 0.20) were carried on as covariates in the multivariate cox proportion hazard analysis. The beta coefficients from the model were used to build a point-based scoring system. Clinical impact was assessed using decision curves.

## INTRODUCTION

Cancer is a common disease among the elderly, as two-thirds of newly diagnosed cancers and three-quarters of cancer-related deaths occur after 65 years of age in Western countries [1]. However, older cancer patients are usually excluded from clinical trials, mainly because of comorbidities and functional impairment [2, 3]. Data from evidence-based-medicine is thus lacking for therapeutic decisions in this population, and one of the main issues is to avoid situations of over- and under-treatment, and to provide guidance for clinicians in the decision to instate exclusively supportive care [4, 5]. Cancer treatment decisions in this setting mainly rely on the Geriatric Assessment (GA) recommended by the International Society of Geriatric Oncology (SIOG) [6]. By assessing patient heterogeneity in terms of social environment, comorbidities, dependency, nutrition, mobility, mood and cognition, the GA detects vulnerabilities that are linked to poor outcomes and treatment complications [6]. In particular, impaired mobility is an independent predictor of early death among older cancer patients [7], assessed among other things by walking speed (also named gait speed) over a short distance of 4 meters [8]. This is a simple clinical and geriatric tool, easy to implement in daily oncological practice, and we have previously shown that a slow gait speed is significantly associated with geriatric impairments, which are in turn predictive of early death at 6 months [8, 9]. In contrast, the feasibility of administration of the GA depends on the local availability of geriatric expertise. Four clinical and biological scores predictive of early death alongside some items from the GA have been suggested, but they lack of simplicity, and thus remain difficult to implement in daily practice [10–13].

In this study, we propose a simple score with five clinical items to predict 6-month mortality risk among older cancer patients, and to guide therapeutic decisions.

## RESULTS

### Patients and their baseline characteristics

**Figure 1:**
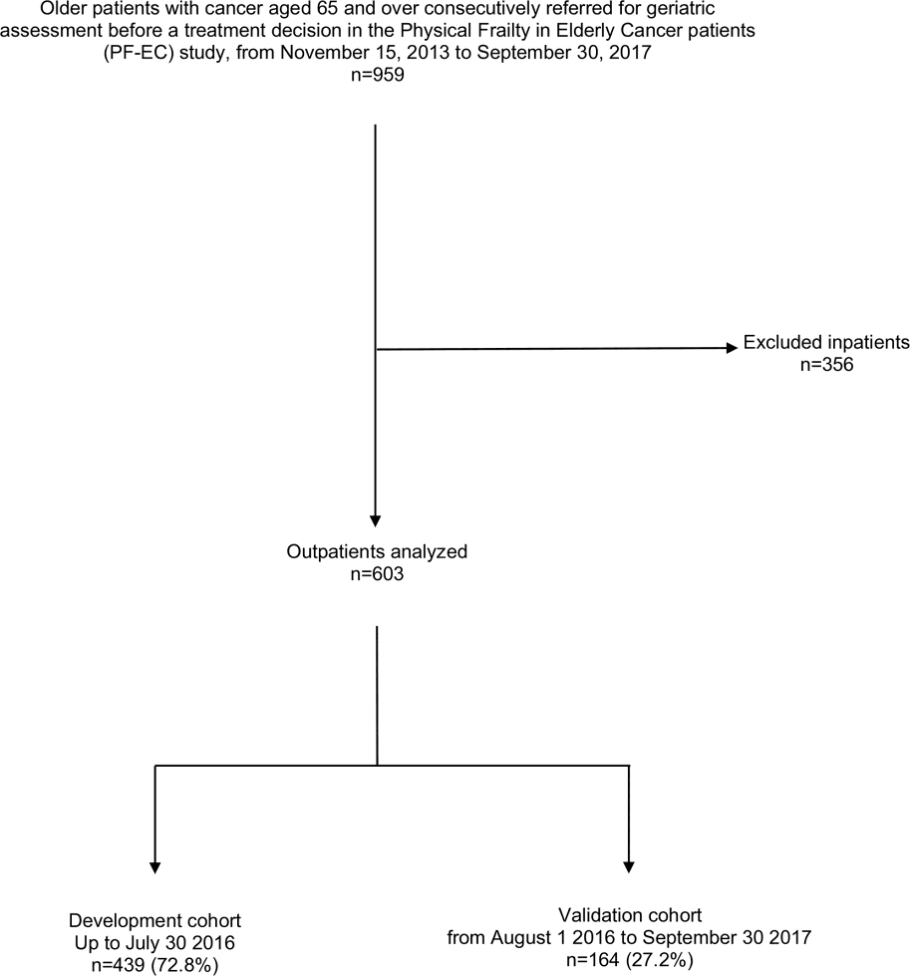
**Consort diagram for the patient selection.**

Among the 959 consecutive older cancer in- and out-patients from the PF-EC cohort study, aged ≥ 65 years who were referred for GA up to September 30 2017, we excluded 356 inpatients. A total of 603 outpatients were included, 439 patients for the development cohort, and 164 for the validation cohort (Figure 1).

In the whole cohort, the mean age was 81.2 ± 6.1 years. The mean time lapse between the initial oncology consultation and the geriatric oncology consultation was 6.6 days ± 2.0. Most patients were West European (62.7%), women (53.5%), and had locally advanced (38%) or metastatic cancers (45%). As expected, colorectal, breast and lung cancers were the most common cancer types, whereas prostate cancer was uncommon because of the absence of urological departments in our two centers (3%). The Frailty phenotype concerned 58% of the overall cohort [14]. The geriatric domains impaired concerned 13.5% (BMI < 21 kg/m^2^) to 67.5% (grade 3 and/or 4 comorbidities) of the sample according to the measures and thresholds used (Table 1, Supplementary Table 1).

**Table 1:** Baseline characteristics of 603 older patients with cancer.

**Variables**	**Development cohort n=439**	**%**	**Validation cohort n=164**	**%**	P*
**Age (y)**					.5
65-76	100	23	39	24	
77-81	114	26	44	27	
82-85	111	25	40	24	
86-103	114	26	41	25	
**Sex (men)**	213	48.5	66	40.2	.06
**Cancer site**					.2
Colorectal	78	17.8	31	19	
Breast	75	17.1	30	18.3	
Lung	68	15.5	24	14.6	
Liver	60	13.7	25	15.2	
Digestive non-colorectal**	58	13.2	21	12.8	
Genito or Urinary	33	7.5	7	4.2	
Hematological	23	5.2	11	6.7	
Skin with melanoma	13	3.0	3	1.8	
Prostate	12	2.7	4	2.4	
Other†	19	4.3	8	5	
**Cancer extension**					.1
Local	82	19	22	13	
Locally-advanced	158	36	70	43	
Metastatic	199	45	72	44	
**Decision to instate exclusively supportive care**	86	19.5	38	23	.3
**ECOG-PS ≥ 3**	131	30	73	44.5	**.007**
**Patients living alone**	181	41	60	36.5	.2
**Comorbidities (CIRSG):**					
Total ≥ 14	217	49	103	63	**.003**
Number of grade 3 and/or 4	307	70	100	61	.03
**Dependency**					
ADL ≤ 5/6	146	33	58	35	.4
IADL ≤ 3/4	284	65	102	62	.5
**Nutrition**					
BMI <21 kg/m^2^	53	12	29	18	.08
Unintentional weight loss ≥5% (yes)	225	51	67	41	**.01**
**Mobility**					
Gait Speed < 0.8 m/s	242	55	103	63	.06
**Depressed mood**					
Mini GDS ≥ ¼	195	44	66	40	.3
**Cognition**					
MMSE < 24/30	166	38	50	30	**.04**

*chi-square test or Fisher’s exact test for categorical variables as appropriate; ** Oesophagus (n=9), gastric (n=20), pancreas (n=25), bile duct (n=15), duodenum (n=3), anus (n=3), gastro intestinal and stromal tumours (GIST) (n=4); † Sarcoma (n=5), mesothelioma (n=8), unknown primary site (n=10), head and neck (n=3), thymus (n=1); ECOG-PS: Eastern Cooperative Oncology Group Performance Status; CIRS-G: Cumulative Illness Rating Scale Geriatric; ADL: Activity of Daily Living; IADL: Instrumental ADL; BMI: Body Mass Index; Mini-GDS: Mini Geriatric Depression Scale; MMSE: Mini Mental State Examination.

There was no difference between the development and validation subsets for cancer-related and demographic data. Significant differences were found for comorbidities, weight loss, and cognitive impairment. There was a larger proportion of patients presenting weight loss (≥ 5%), cognitive impairment and overall co-morbidities in the development cohort, while there were more severe comorbidities in the validation cohort (Table 1).

### Development of a multivariate prediction model for 6-month mortality

**Figure 2:**
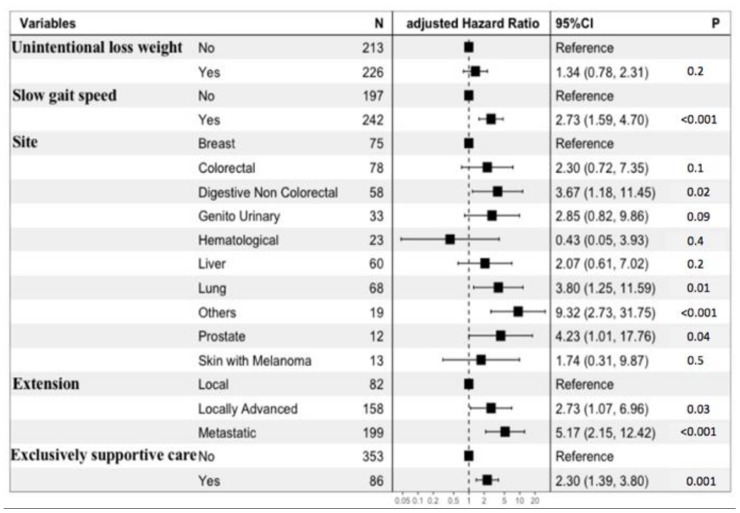
**Forest plot of the multivariate prediction model.**

**Figure 3:**
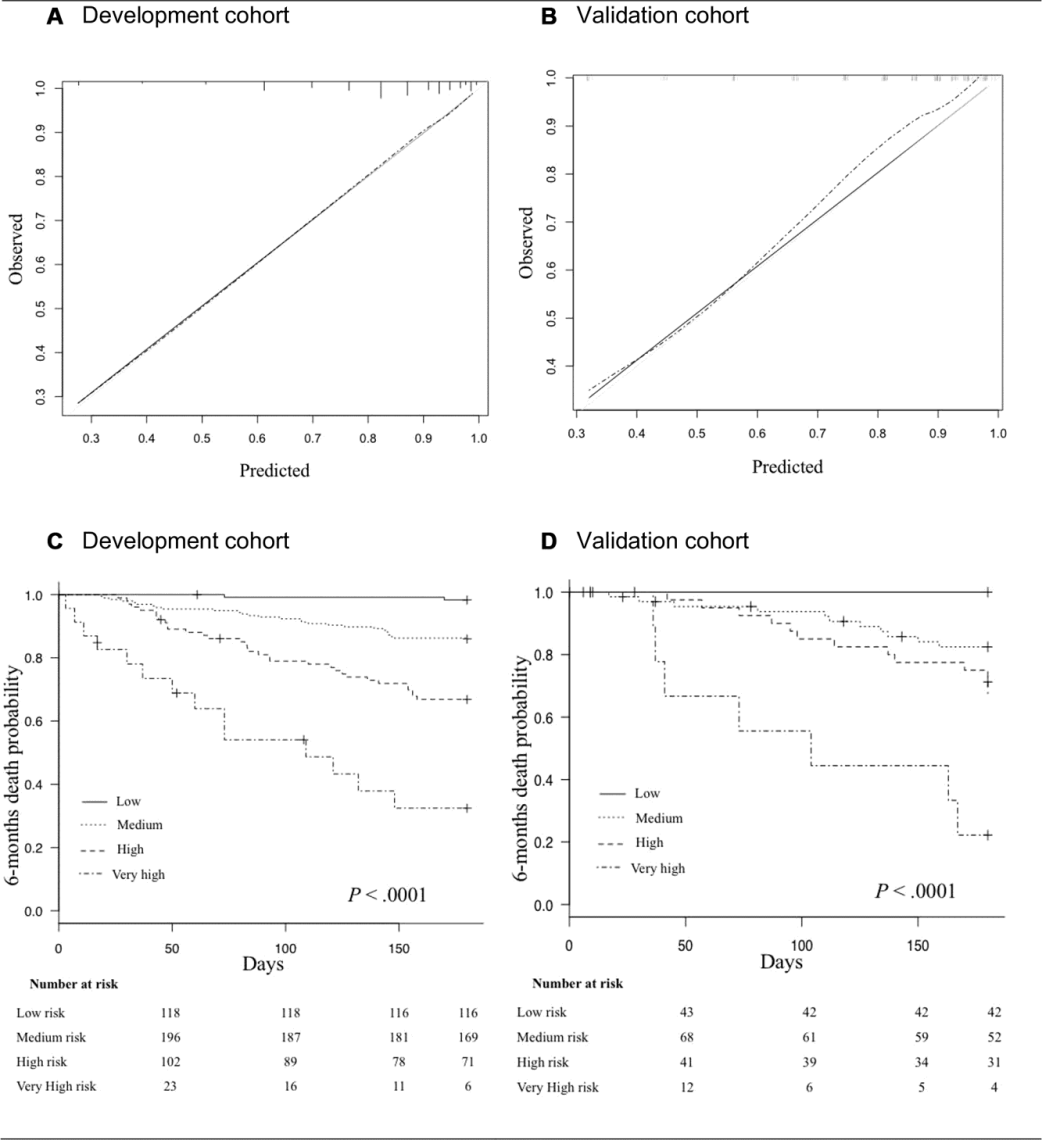
(**A**, **B**) Calibration curves in the development cohort (**A**), and in the validation cohort (**B**). The grey line indicates the ideal prediction; The black line indicates prediction with the GRADE; The dashed line indicates prediction with optimism correction. (**C**, **D**) Kaplan Meyer survival curves for 6-month mortality according to risk-groups in the development cohort (**C**), and in the validation cohort (**D**).

At 6 months, the mortality rate was 17.5% (n=77/439). In univariate analysis, male gender, cancer site, cancer extension, exclusively supportive care, malnutrition (BMI < 21 kg/m^2^ and weight loss ≥ 5%), and impaired mobility (gait speed < 0.8 m/s) were significantly associated with 6-month mortality. Age, comorbidities, dependency, depressed mood and cognitive impairment were not associated with 6-month mortality (Table 2). The final multivariate prediction model included two geriatric variables, malnutrition (unintentional weight loss) and impaired mobility (slow gait speed); two oncological variables (cancer site, cancer extension), and the decision whether or not to instate exclusively supportive care (Figure 2, Table 3). There was no interaction between cancer site, cancer extension, and exclusively supportive care. The final scores ranged from 2 to 19 with a median of 9 (6-11). Four groups at increasing risk were identified: 118 patients (25%) were at low risk (2 to 6), 196 (45%) at medium risk (7 to 10), 102 (23%) at high risk (11 to 14), and 23 (5%) at very-high risk (15 and over). Overall, the risk of early death ranged from 2% to 61%. The score was well calibrated (Figure 3A), and discrimination was good, with a Harrell’s C index of 0.75 (0.69-0.81). The Kaplan-Meier plot showed significant discrimination across the four risk groups. In particular, the 6-month risk of death was 2%, 14%, 32% and 61% for the low, medium, high and very high-risk groups respectively (Figure 3C).

**Table 2:** Univariate predictors of 6-month mortality in the 439 older patients with cancer in the development cohort.

**Variables**	**Non-survivors** **n= 77**	**%**	**Survivors** **n= 362**	**%**	**Non-adjusted** **HR [95% CI]**	P*
**Age (y)**						.5
65 to 76	17	22	83	23	1 (reference)	
77 to 81	22	29	92	25	1.2 [0.6-2.2]	
82 to 85	15	19	96	27	0.8 [0.4-1.6]	
86 to 103	23	30	91	25	1.3 [0.7-2.4]	
**Sex (men)**	46	59.7	168	46.4	1.7 [1-2.6]	.03
**Cancer site**						**<.0001**
Breast	4	5.2	71	20	1 (reference)	
Colorectal	11	14.3	67	19	2.8 [0.9-8.7]	
Lung	18	23.4	50	14	5.7 [1.9-16.8]	
Liver	8	10.4	52	14	2.6 [0.8-8.8]	
Digestive non-colorectal**	14	18.2	44	12	5.1 [1.7-15.6]	
Genito or Urinary	7	9	26	7	4.2 [1.2-14.5]	
Hematological	1	1.3	22	6	0.8 [0.1-7.3]	
Skin with melanoma	2	2.6	11	3	3.4 [0.6-18.6]	
Prostate	4	5.2	8	2	6.8 [1.7-27.0]	
Others †	8	10.4	11	3	11.4 [3.4-37.9]	
**Cancer extension**						**<.0001**
Local	6	8	76	21	1 (reference)	
Locally-advanced	21	27	137	38	1.8 [0.7-4.5]	
Metastatic	50	65	149	41	3.8 [1.6-8.8]	
**Decision to instate exclusively supportive care**	26	34	60	16.5	2.5 [1.5-4.0]	**.0001**
**ECOG ≥ 3**	35	45	96	26.5	2.2 [1.4-3.5]	**.0005**
**Patients living alone**	31	40	150	41	1.0 [0.7-1.4]	.9
**Comorbidities (CIRS-G)**						
Total ≥ 14	37	48	142	39	1.4 [0.9-2.2]	.1
Number of grade 3 and/or 4	19	25	113	31	1.3 [0.8-2.3]	.2
**Dependency**						
ADL ≤ 5/6	32	41.5	114	31.5	1.5 [1.0-2.4]	.06
IADL ≤ 3/4	56	73	228	63	1.5 [0.9-2.5]	.1
**Nutrition**						
BMI < 21 kg/m^2^	16	21	40	11	2.0 [1.1-3.4]	.01
Unintentional weight loss ≥5%	52	67. 5	174	48	2.0 [1.2-3.3]	**.002**
**Mobility**						
Gait Speed < 0.8 m/s	58	75	184	51	2.7 [1.6-4.6]	**.0001**
**Depressed Mood**						
Mini-GDS ≥ 1/4	37	48	158	44	1.2 [0.8-1.9]	.4
**Cognition**						
MMSE < 24/30	45	58	182	50	1.4 [0.9-2.0]	.1

*Log rank test; HR: hazard ratio; ** Oesophagus (n=9), gastric (n=20), pancreas (n=25), bile duct (n=15), duodenum (n=3), anus (n=3), gastro intestinal and stromal tumours (GIST) (n=4); † Sarcoma (n=5), mesothelioma (n=8), unknown primary site (n=10), head and neck (n=3), thymus (n=1); ECOG-PS: Eastern Cooperative Oncology Group Performance Status; CIRS-G: Cumulative Illness Rating Scale Geriatric; ADL: Activity of Daily Living; IADL: Instrumental ADL; BMI: Body Mass Index; Mini-GDS: Mini Geriatric Depression Scale; MMSE: Mini Mental State Examination.

**Table 3:** Multivariate predictors of 6-months mortality and scoring system based on the development cohort of 439 patients.

**Variables**	**Beta-coefficient**	**Standard error**	**Scoring**
**Unintentional loss weight ≥ 5%**	0.2	0.2	1
**Gait speed < 0.8 m/s**	1.0	0.2	3
**Cancer site**			
Breast	Reference	Reference	0
Hematological	-0.8	1.1	-3
Colorectal	0.8	0.5	3
Liver	0.7	0.6	2
Skin with melanoma	0.5	0.8	2
Genito or Urinary	1.0	0.6	3
Digestive non-colorectal	1.3	0.5	4
Lung	1.3	0.5	4
Prostatic	1.4	0.7	5
Others	2.2	0.6	7
**Cancer extension**			
Local	Reference	Reference	0
Locally-advanced	1.0	0.4	3
Metastatic	1.6	0.4	5
**Decision to instate exclusively supportive care**	0.8	0.2	3

### Internal validity and clinical impact of the final multivariate prediction model

In the validation cohort, the 6-month mortality rate was 18.9% (n=31/164). The score ranged from 0 to 18 with a median of 9 (6-11). 43 patients (26%) were at low risk, 68 (41%) at medium risk, 41 (25%) at high risk, and 12 (7%) at very high-risk. Overall, the risk of early death ranged from 0% to 58%. The score was also well calibrated (Figure 3B) and discrimination was good, with a Harrell’s C index of 0.76 (0.66-0.86), close to that for the development cohort. The Kaplan-Meier plot showed significant discrimination across the four risk groups (Figure 3D).

In stratified analyses, discrimination showed consistent results, with the best Harrell’s C index at 0.81 (0.63-0.98) in the 77- to 81-year-old subset (Table 4).

**Table 4:** Discrimination of the GRADE in the validation cohort.

**Subgroups**	**N° of patients (n)**	**Harrell’s C index [95% CI]**
**Age (y):**		
65-76	(n=39)	0.78 [0.5-1.0]
77-81	(n=44)	0.81 [0.6-0.9]
82-85	(n=40)	0.66 [0.4-0.8]
86-103	(n=41)	0.79 [0.5-0.9]
**Cancer extension:**		
Non-metastatic	(n=92)	0.77 [0.6-0.9]
Metastatic	(n=72)	0.70 [0.5-0.8]
**Functional status:**		
ECOG-PS < 3	(n=91)	0.76 [0.6-0.9]
ECOG-PS ≥ 3	(n=73)	0.73 [0.6-0.8]

ECOG-PS: Eastern Cooperative Oncology Group Performance Status.

**Figure 4:**
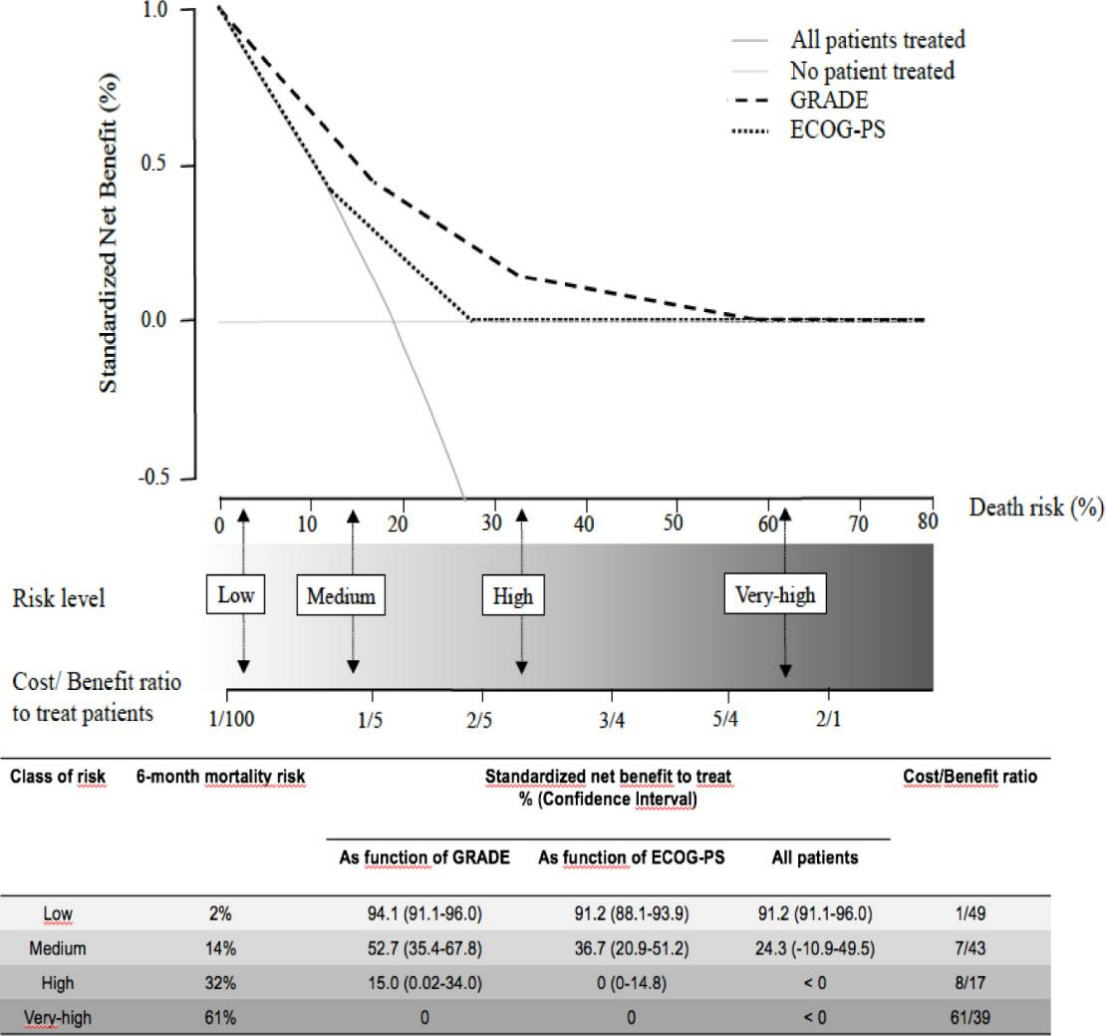
**The decision curves show estimates of the SNB (%) over a range of probability thresholds used to categorize observations as “high risk”.** The curves help to evaluate a treatment policy that recommends treatment for patients who are considered to be “high risk” by comparing the population impact of a risk-based policy to “treat all” (cross line in grey) and “treat none” (baseline) intervention policies. A model for prediction of early death (curves in black) according to ECOG-PS, and the GRADE. At a given risk threshold for early death, the graph gives the expected SNB per patient for “treat none”, “treat all”, and to treatment according to the ECOG-PS, and the GRADE, in relation to the related Cost/Benefit (C/B) ratio.

The decision curves provided better performances for our scoring system than for the ECOG-PS in accurately identifying the risk thresholds for purpose of therapeutic-decisions (Figure 4). In particular, at the 2% and 14% risk thresholds for early death our scoring system points the need for cancer treatment and is related to a favorable C/B ratio among older patients. At the 32% risk threshold for early death our scoring system yields a less favorable C/B ratio for cancer treatment among older patients and suggests the need for caution in therapeutic decisions (tailored case by). At 61% risk threshold for early death our scoring system discourages cancer treatment among older patients, since the C/B ratio is not in favor of these patients. One the basis of these considerations, 23/38 (60%) patients in the exclusively supportive care subset would be less likely to be undertreated. Conversely, 26/126 (21%) patients in the active treatment subset would be less likely to be overtreated. Overall, according to the GRADE, 49/164 (30%) patients would be misclassified for the final therapeutic decision.

### Practical clinical application

**Figure 5:**
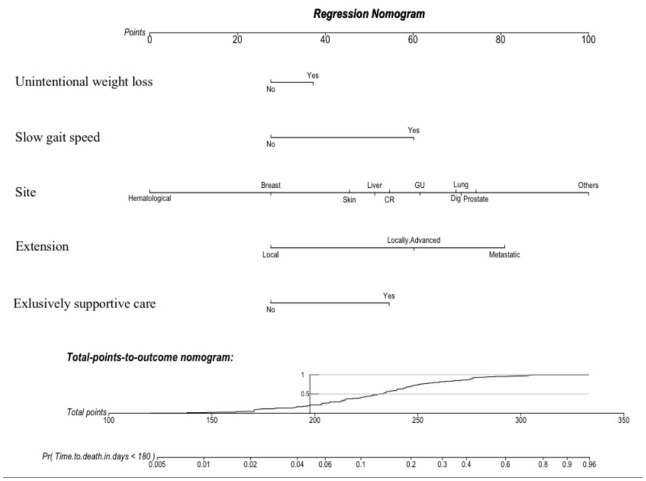
**A nomogram is a graphical calculating device based on the results of a multivariate Cox regression.** It is a quick way to interpret the 6-month mortality risk predicted by the multivariate Cox model. A numerical scale was created allocating scores for each predictor: Unintentional weight loss (“Yes”: 37; “No”: 28); Slow gait speed (“Yes”: 60; “No”: 28); Site (Hematological: “0”; Breast: “28”; Skin: “46”; Liver: “51”; CR: “55”; GU: “62”; Dig: “70”; Lung: “71”; Prostate: “74”; Other: “100”); Extension (Local: “28”; Locally-Advanced: “60”; Metastatic: “81”); and Exclusively supportive cares (“Yes”: 55; “No”: 28). The total score derived from all the covariates ranges from 112 to 333 points and indicates the probabilities (*Pr*) of dying in the 6-month follow-up. This device is available on the website: https://grade.shinyapps.io/dynnomapp/ Skin: skin with melanoma; CR: colorectal; GU: genito-urinary; Dig: digestive non-colorectal**.**

For therapeutic decision purposes, the GRADE should be used in a two-step approach: the sum of the first four variables (i.e. weight, gait speed, cancer site and extension) gives a risk of death at baseline; then the addition of the last variable enables the final decision on whether or not it is exclusively supportive care that is required. This dynamic process is expected to help with weighing up therapeutic decisions, to avoid under- and over-treatment situations. We also created a nomogram using the five clinical variables (Figure 5) with a free website link to be used by clinicians in their daily practice: https://grade.shinyapps.io/dynnomapp/. In brief, the website gives the patient’s expected survival by way of click-check variables.

## DISCUSSION

Here, we provided a simple geriatric score namely GRADE to predict 6-month mortality, and to guide oncologists in their therapeutic decisions.

In our study, for all 603 patients in the two cohorts, therapeutic decisions were made in a multidisciplinary consultation meeting, which reflects the real-life situation of older patients with cancer.

Our score is composed of five clinical variables, including cancer site and extension, non-intentional weight loss ≥5%, gait speed <0.8 m/s and the decision or not to instate exclusively supportive care. This is the main strength of our simple score, since it is easy to perform in daily oncological practice with only a few additional minutes to a normal consultation. Particularly, in our experience, the mean time to measure gait speed is 69.5 seconds, ideally measured during the time spent between the waiting room and the consultation room. In addition, we found that gait speed < 0.8 m/s was an independent, strong covariate in our scoring system. A slow gait speed is a well-known predictive factor for mortality among older adults, and it is strongly associated with frailty and treatment complications [8, 14–16]. We have previously shown that a gait speed <1m/s is significantly associated with at least one geriatric impairment on the GA, and thus takes the heterogeneity of ageing with cancer into account [18]. A slow gait speed was also found to be independently associated with early death in the recently published Nice Cancer Ageing Survival (NCAS) score [13]. This score predicts early death at 100 days for older cancer patients, but it is not feasible in daily practice since it uses the mini nutritional assessment (MNA) instead of weight loss as the nutrition parameter. In a first oncological consultation, MNA scoring would require up to 15 additional minutes [17]. A limitation of our score could be its poorer discrimination compared to the NCAS score [13]. However, we chose a compromise between feasibility in daily practice, and the predictive performances of a survival score of this type. In addition to the NCAS score, other scoring systems have been specifically developed for older cancer patients for the prediction of longer-term mortality. (Table 5).

**Table 5:** Comparison of baseline characteristics and predictive performances of scoring systems for mortality designed for older patients with cancer.

**Scoring systems**	**Number of patients Ethnicity**	**Age (y) Median age (range)**	**Outcome (mortality rate)**	**Variables**	**Discrimination, C index (95%CI) Very good (VG) Good (G) Moderate (M)**	**Advantages**	**Disadvantages**
**CGA-based score 2011 [10]**	n=249 Asian	≥70 77 (70-94)	1-, 2-, and 3-year (69%)	Total = 6 Age, Albumin, GDS, DETERMINE nutritional index, ECOG-PS, cancer-extension	Harrell’s C index * 0.71(NA) G	Short to long term survival estimation Internal validity	Time to scoring with biological variable (1 day at least) Asian population only
**Onco-MPI 2016 [11]**	n=658 Not reported	≥70 77 (70-96)	1-year (17.4%)	Total = 12 Age, sex, BMI, ADL, IADL, ECOG-PS, CIRSG, number of drugs, MMSE, caregivers, cancer-site, cancer-extension	Harrell’s C index 0.86 (0.84-0.89) ** VG	The best discrimination	No validation Time to scoring based on geriatric assessment (45 min at least)
**MNA-based sore 2016 [12]**	n=606 Not reported	≥70 NA	1-year (37%)	Total = 4 MNA-modified version, cancer-site, cancer-extension, lymphocytes.	AUC 0.69 (NA) † M	External validity	Time to scoring with biological variable (1 day at least) Moderate discrimination
**NCASS 2018 [13]**	n=1050 Not reported	≥70 81.8 (70-100)	100-day (20%)	Total = 5 MNA-full version, Gait speed, ECOG-PS, cancer-site, cancer extension	AUC 0.79 (0.76-0.83) * G	Internal validity Good discrimination Large sample	Time to scoring with MNA-full version (15 min at least)
**GRADE 2019**	**n=603 Various ethnicity**	**≥ 65 82 (65-103)**	**6-month (17.5%)**	**Total = 5 Weight loss, Gait speed, cancer-site, cancer-extension, exclusively supportive cares**	Harrell’s C index 0.75 (0.65-0.84) * G	**Internal validity Good discrimination Various ethnicity Time to scoring (3 min at most) Cost/benefit ratio to treat**	**External validity missing**

*internal validity; **no validation; † external validity; ADL: Activity of Daily Living; BMI: Body Mass Index; CGA: Comprehensive Geriatric Assessment; CIRSG: Cumulative Illness Rating Scale Geriatric; DETERMINE: Disease Eating poorly Tooth loss/mouth pain Economic hardship Reduced social contact Multiple medicines Involuntary weight loss/gain Needs assistance in self-care Elder years > 80; ECOG-PS: Eastern Cooperative Oncology Group Performance Status; GRADE: onco-Geriatric scoRe of eArly DEath; GDS: Geriatric Depression Scale; IADL: Instrumental-ADL; MMSE: Mini Mental State Examination; MNA: Mini Nutritional Assessment; NCASS: Nice Cancer Ageing Survival Score; Onco-MPI: Oncologic Multidimensional Prognostic Index.

Another strength of our study is the robust methodology that we used as recommended for the development and validation of a multivariate prediction model (TRIPOD guidelines) [18]. We prospectively enrolled patients and assessed development and internal validity with a non-random split sample of patients. We used a Cox proportional hazard regression model that took into account the time to death and selected the five variables to maximize the likelihood. We validated the clinical impact of our score with decision curves, and we showed better performances than the ECOG-PS for cancer treatment decisions, since it is an unreliable clinical scale in older cancer patients [19]. Our score could also be useful for risk adjustment when a clinical trial on cancer is to be performed among older patients. Finally, our score could be useful in epidemiological studies to compare study populations, and patient outcomes across different health-care organizations.

Our population is ethnically diverse and the population characteristics are similar to those in other large studies on older cancer patients (*e.g.* ONCODAGE and ELCAPA) [7, 20].

Among older cancer patients, the therapeutic decision is a complex process that relies on multidisciplinary expertise, cancer-related symptoms, the specific type of treatment for the cancer, and the patient’s preference, so that a prognostic score cannot be the sole component in the decision-making process. Our score provides three levels of information for the clinician: the risk of death linked to geriatric health (i.e. weight loss and gait speed) and to the cancer extension; the benefit of treating according to the GRADE (i.e. life extended); and the treatment cost in terms of morbidity (i.e. side effects). Thus, our score accurately identifies four groups at risk for death and increasing cost. Moreover, the originality of the GRADE is that it takes into account the decision whether or not to choose exclusively supportive care for a patient. This is particularly important for therapeutic decision-making, in metastatic settings, when the question of whether to treat or not is a daily challenge to avoid under- or over-treatment of older cancer patients. It is now well-known that older cancer patients can be under-treated, usually with sub-optimal-dose treatments if they are not too frail [21, 22]. They can also be over-treated, particularly if exclusively supportive care should be preferred. Our score is helpful in that it contributes to the therapeutic decision using the variable “exclusively supportive care”. This is particularly true for medium risk groups for whom a “supportive care” decision would put them in a high-risk group, and therefore a missed chance. We are aware that breaking bad news such as the decision to instate exclusively supportive care (i.e. treatment of symptoms) is difficult for the patient but also for the oncologist [23]. However, even a less aggressive chemotherapy regimen is potentially morbid, and usually non-beneficial in this setting (i.e. metastatic cancer patients with a high or very-high risk according to the GRADE) [24, 25].

Let us consider the real case of an 87-year-old man with metastatic lung cancer with a 15% weight loss, and a gait speed ≥ 0.8 m/s. Thus, the GRADE is 10 (medium risk), with a 6-month mortality risk of 14%. If this patient is offered exclusively supportive care, he would be up-graded to 13 in the high-risk group, meaning decreased survival and increased cost. For this reason, he should be offered chemotherapy. Now, let us look at the same patient with a gait speed < 0.8 m/s. Before any treatment decision, the GRADE is 13 (high-risk), with a 6-month mortality risk of 32% and an unfavorable cost/benefit ratio. Even low-dose chemotherapy would lead to a greater cost and morbidity for a small benefit. The decision to instate exclusively supportive care should thus be preferred in this clinical situation.

We encourage active treatment for patients with an estimated risk of early death up to 14%, since in this situation, there is a cost/benefit ratio in favor of active treatment.

## CONCLUSION

In this study, we developed and validated a simple score easy to implement in daily oncological practice, to predict early death among older cancer patients and to guide oncologists in their treatment decisions.

## MATERIALS AND METHODS

### Reporting

We followed the Transparent Reporting of a multivariate prediction model for Individual Prognosis Or Diagnosis (TRIPOD) guidelines [18].

### Study design and population

Patients were recruited from the Physical Frailty in Elderly Cancer patients (PF-EC) cohort. This prospective, observational two-center cohort study started in November 2013, and is described in detail elsewhere [9]. Briefly, all consecutive older in- and out-patients referred for a geriatric oncology assessment were prospectively included in a registry when a diagnosis of cancer was established and when a frailty was suspected, before any cancer treatment decision.

For the present study, we analyzed all outpatients who presented up to September 30 2017, regardless of tumor site or stage. We excluded inpatients because of the impracticality of conducting mobility tests (e.g. infusions limiting walking measures). The inclusion date was the date of the first geriatric oncology visit.

Informed consent was obtained from the patients before inclusion. The study was approved by the local ethics committee (CLEA-2015-019, Avicenne Hospital, Bobigny, France).

### Cancer-related and demographic data

Demographic data (age, sex, region of origin classified as follows: West Europeans, East Europeans, Latin Europeans, North Africans, Sub-Saharan Africans, and Asians), tumor characteristics (site, extension: local, locally advanced *i.e.* unresectable tumor with no distant metastases, or metastatic disease *stricto sensu*) and Eastern Cooperative Oncology Group Performance Status (ECOG-PS) were obtained at the first geriatric oncology visit as part of the GA. Cancer treatment modes were categorized as exclusively supportive care or not, and were prospectively collected in the 6-month follow-up according to the treatment finally administered (i.e. supportive care only vs. at least one of the following treatment modes: surgery, chemotherapy, targeted therapy, hormonotherapy, radiotherapy, percutaneous treatment, and intra-arterial treatment). All treatment regimens, including exclusively supportive care, were validated in weekly institutional multidisciplinary meetings.

### The geriatric assessment (GA)

The GA was performed at the first geriatric oncology visit and included seven domains (social environment, comorbidities, functional status, mobility, nutrition, mood, and cognition) [8, 14, 26–31] (Table 6).

**Table 6:** The geriatric assessment in the PF-EC cohort.

**Domains**	**Tools**	**Range**	**Abnormal if**	**Reference**
Social environment	Question: Are you living alone?	-	Yes	-
Comorbidities	CIRSG No. of grade 3 (severe) No. of grade 4 (very severe)	0-56 - -	≥ 14 (median) ≥ 1 ≥ 1	[26]
Functional status	ADL IADL	0-6 0-4	≤5 ≤ 4	[27] [28]
Mobility	GS (m/s)	-	< 0.8	[8]
Nutrition	BMI (kg/m^2^) Unintentional weight loss in the previous year (%)	- -	< 21 ≥ 5	[29] [14]
Mood	Mini-GDS	0-4	≥ 1	[30]
Cognition	MMSE	0-30	< 24	[31]

CIRSG: Cumulative illness Rating Scale Geriatric; ADL: Activity of Daily Living; IADL: Instrumental ADL; GS: gait speed; BMI: Body Mass Index; Mini-GDS: mini Geriatric Depression Scale; MMSE: Mini Mental State Examination.

### Outcome

Overall 6-month mortality following the GA was recorded to assess predictors of early death. Vital status was determined from medical records or by telephoning patients or their families.

### Statistical analyses

We allocated patients included up to July 30 2016 to the development cohort, and the remaining patients up to September 30 2017 to the validation cohort.

Categorical data are expressed as numbers and proportions, and continuous data as means and standard deviation (SD) or medians and quartiles (25^th^-75^th^).

Comparisons of baseline characteristics (cancer, demographic data, and GA-components) between the development and validation subsets were performed using the chi-square test or Fisher’s exact test for categorical variables, as appropriate.

We performed a correlation assessment using the Spearman’s rho test as appropriate for categorical variables. Multicollinearity between variables was defined as a rho test value ≥ 0.50.

### Development of the onco-Geriatric scoRe of eArly DEath (GRADE)

A comparison of baseline characteristics (cancer, demographic data, GA-components) between six-month survivors and non-survivors in the development subset was performed using the log rank test. Univariate predictors of six-month non-survival were expressed using non-adjusted Hazard Ratios (HR) and 95% confidence intervals (95%CI). Variables yielding *P* values under 0.20 in the univariate analysis were considered for inclusion in the multivariate analysis. Because of multicollinearity between the GS and the IADL, the ADL and the ECOG-PS, we choose GS as a covariate of functional status. We therefore created a Cox multivariate proportional-hazard regression model to assess multivariate factors associated with 6-month mortality. Multivariate predictors were expressed by the beta-coefficient with standard error, and by adjusted HR (aHR) with 95%CI. Graphically, multivariate predictors were presented in a forest-plot. Model assumptions were verified. To maximize likelihood while minimizing losses in prediction, a final multivariate prediction model was created with a backward selection procedure according to the lowest Akaïke Information Criterion (AIC) [32]. A scoring of each predictor was performed using Schneeweiss’s beta-coefficient point-based scoring system [32]. This scoring system weights by 1 unit more or less with each 0.3 increase or decrease in the beta-coefficient. We categorized this score into four groups of increasing risk on the basis of the lowest AIC value in Cox regression. Graphically, we assessed the calibration slope in the final multivariate prediction model. Discrimination by the final multivariate prediction model was assessed using Harrell’s C index with 95%CI. Given the current lack of consensus on the best threshold to consider for the quality of discrimination for survival prediction models, we used a previously described categorization of Harrell’s C index discrimination as well: 0.5-0.59 (poor), 0.6-0.69 (moderate), 0.7-0.79 (good), 0.8-0.89 (very good), and ≥ 0.90 (excellent) [33]. Survival curves were plotted according to the Kaplan-Meier method with the final score divided into four groups.

### Internal validation

we assessed the calibration and discrimination of the final score in the validation subset with the same methods as described above. Stratified discrimination analyses were performed in age quartiles, for an ECOG-PS ≤ 2 *vs* > 2, and for non-metastatic *vs* metastatic cancers. To assess the clinical impact of our score, we used the standardized net benefit (SNB) derived from decision curves [34, 35] (Supplementary Methods).

All tests were two-sided, and the threshold for statistical significance was set at *P*<0.05. The data was analyzed using R statistical software (version 3.4.3, R Foundation for Statistical Computing, Vienna, Austria; http://www.r-project.org). A multivariate imputation by chained equations was used to handle missing data for weight loss (n=9), and GS (n=3), via the MICE package in R.

## Supplementary Material

Supplementary Methods

Supplementary Table 1
