# Supplementary material for: Development, validation and clinical impact of a prediction model for 6-month mortality in older cancer patients: the GRADE
Source: Aging (Albany NY). 2020 Mar 10;12(5):4230–46. doi: 10.18632/aging.102876 (PMC7093177; doi:10.18632/aging.102876)
Supplement: Supplementary Methods [file aging-12-102876-s001..pdf]

SUPPLEMENTARY MATERIAL

METHODS

The SNB was first developed to assess the potential population-level impact of incorporating a risk prediction instrument into clinical practice. The assumption is that some patients will experience the outcome (*i.e.* cases), and some patients will not (*i.e.* controls). The SNB is defined in terms of true and false positive rates, outcome prevalence, and risk threshold. Thus, the interpretation of the SNB value depends on the risk threshold considered - 6-month mortality, in the present study. The SNB is closely related to a Cost/Benefit (C/B) ratio in the treatment of patients. Here, we assume that treating a control has a cost (C) (*e.g.*, side effects of treatment), whilst there is some expected benefit (B) in treating a case (*e.g.*, life extended, morbidity reduced). As we identified four increasing risk categories for early death (*i.e.*, 2%, 14%, 32%, and 61%), we analyzed the SNB (95% CI), and C/B at these thresholds. We then compared the decision curves with ECOG-PS, one of the most widely used measures for therapeutic decisions in oncology.
