# Supplementary material for: Development, validation and clinical impact of a prediction model for 6-month mortality in older cancer patients: the GRADE
Source: Aging (Albany NY). 2020 Mar 10;12(5):4230–46. doi: 10.18632/aging.102876 (PMC7093177; doi:10.18632/aging.102876)
Supplement: Supplementary Table 1 [file aging-12-102876-s002..pdf]

SUPPLEMENTARY TABLE

Supplementary Table 1. Baseline ethnic and treatment characteristics of 603 older patients with cancer.

Variables	Development cohort n=439	%	Validation cohort n=164	%	*P**
**Region of origin:**					.9
West European	277	63.2	101	61.6	
East European	73	16.6	28	17	
Latin European	20	4.6	8	5	
North African	45	10.2	17	10.3	
Sub-Saharan African	12	2.7	7	4.1	
Asian	12	2.7	3	2	
**Cancer-treatment modalities:**					.1
Chemotherapy	155	35	45	27	
Surgery	125	28.5	47	28.6	
Radiotherapy	104	24	27	16	
Hormonotherapy	75	17	21	13	
Targeted therapy	33	7.5	10	61.6	
Percutaneous treatment (liver)	32	7.5	9	5	
Intra-arterial treatment (liver)	14	3	8	5	

*chi-square test or Fisher's exact test for categorical variables as appropriate.
